# 2025 Chinese guidelines for the diagnosis and treatment of systemic lupus erythematosus

**DOI:** 10.1515/rir-2025-0017

**Published:** 2025-10-04

**Authors:** Jiuliang Zhao, Shangzhu Zhang, Qian Wang, Xinping Tian, Yaolong Chen, Mengtao Li, Xiaofeng Zeng

**Affiliations:** Department of Rheumatology and Clinical Immunology, Chinese Academy of Medical Sciences & Peking Union Medical College, National Clinical Research Center for Dermatologic and Immunologic Diseases (NCRC-DID), Ministry of Science & Technology, Key Laboratory of Rheumatology and Clinical Immunology, Ministry of Education, State Key Laboratory of Complex Severe and Rare Diseases, Peking Union Medical College Hospital (PUMCH), Beijing, China; Research Unit of Evidence-Based Evaluation and Guidelines, Chinese Academy of Medical Sciences (2021RU017), Evidence-Based Medicine Center, School of Basic Medical Sciences, Lanzhou University, Lanzhou 730000, Gansu Province, China

**Keywords:** systemic lupus erythematosus, diagnosis, treatment, guideline, GRADE

## Abstract

Systemic lupus erythematosus (SLE) is a complex autoimmune disease that poses significant challenges in diagnosis and treatment. In recent years, advances in basic and clinical research related to SLE have led to the emergence of new diagnostic and therapeutic approaches, as well as the continuous updates of international clinical guidelines. Consequently, existing guidelines no longer fully align with current clinical practice in China. Initiated by the National Clinical Research Center for Dermatologic and Immunologic Diseases and the Chinese SLE Treatment and Research Group, and in collaboration with the Chinese Rheumatology Association and Chinese Association of Rheumatology and Immunology Physicians, an expert panel has comprehensively evaluated the latest research evidence and integrated domestic clinical experience. In accordance with the GRADE framework, the “2020 Chinese Guidelines for the Diagnosis and Treatment of Systemic Lupus Erythematosus” have undergone systematic revision. The updated guidelines provide detailed evidence-based recommendations addressing 12 critical clinical concerns prioritized by frontline rheumatologists in China. The revision aims to optimize the scientific rigor of SLE clinical management and enhance patient-centered healthcare services.

## Background

Systemic lupus erythematosus (SLE) is a systemic autoimmune disease characterized by multi-organ involvement, relapsing-remitting course, and the presence of autoantibodies. Without timely treatment, SLE can cause irreversible end-organ damage and fatal outcomes. The pathogenesis of SLE is multifactorial, with contributions from genetic susceptibility, sex-hormonal influences, and environmental exposures (such as viral and bacterial infections).^[1, 2, 3]^ SLE prevalence varies significantly across regions, with global incidence ranging from 1.4 to 11 per 100, 000 individuals. Based on analyses of China’s national health insurance database and the Chinese Rheumatism Data Center (CRDC), the estimated incidence in mainland China is approximately 14.09 per 100, 000,^[4,5]^ with a female-to-male ratio of 10–12: 1.^[6, 7, 8]^ The development of SLE therapeutics has driven substantial improvements in survival outcomes. Epidemiological data reveal a progressive increase in 5-year survival rates from 50%-60% (1950 s) to over 90% (1990 s), with stabilization observed in the 2008–2016 period (95% in high-income *vs*. 92% in low-and middle-income countries).^[9, 10, 11]^ Consequently, SLE has transitioned from an acute, highly fatal disease to a chronic, manageable condition.

Leading international organizations, including the American College of Rheumatology (ACR), the European League Against Rheumatism (EULAR), the British Society for Rheumatology (BSR), and the Asia Pacific League of Associations for Rheumatology (APLAR), have developed their respective guidelines for the diagnosis and treatment of SLE,^[12, 13, 14]^ Similarly, the Chinese Rheumatology Association released two editions of SLE guidelines in 2010 and 2020, ^[15,16]^ which have significantly enhanced the scientific rigor and standardization of clinical decision-making. However, existing guidelines face limitations in guiding current SLE clinical practice in China due to the following reasons: (1) Data from the Chinese SLE Treatment and Research Group (CSTAR) registry cohort demonstrates that the disease onset, clinical manifestations, and prognosis of SLE patients in China differ from those in Western populations.^[6,17,18]^ (2) International SLE management recommendations may not align with China’s clinical realities. (3) With continuous advancements in both SLE therapeutics (including novel agents) and evidence-based guideline methodology, the Chinese 2020 SLE guidelines no longer adequately address current clinical needs. To address these gaps, the National Clinical Research Center for Dermatologic and Immunologic Diseases (NCRC-DID) (Peking Union Medical College Hospital), in collaboration with the CSTAR, the Chinese Rheumatology Association, the Chinese Association of Rheumatology and Immunology Physicians, the Rheumatology Rehabilitation Branch of Chinese Association of Rehabilitation Medicine, and the Chinese Research Hospital Association of Rheumatology and Immunology Professional Committee, has developed the “2025 Chinese Guidelines for the Diagnosis and Treatment of Systemic Lupus Erythematosus (revised edition) “(hereafter referred to as this guideline). This guideline development process adhered to internationally recognized evidence-based medicine standards, while comprehensively considering China’s unique clinical practice and healthcare system characteristics.

## Guideline Development Methods

### Guideline Initiators and Expert Panel

This guideline was initiated by the NCRC-DID (Peking Union Medical College Hospital) and the CSTAR, in collaboration with the Chinese Rheumatology Association, the Chinese Association of Rheumatology and Immunology Physicians, the Rheumatology Rehabilitation Branch of Chinese Association of Rehabilitation Medicine, and the Chinese Research Hospital Association of Rheumatology and Immunology Professional Committee. The development of this guideline was launched on August 21st, 2024, and was finalized on December 21st, 2024.

### Guideline Working Group

A multidisciplinary working group was established, comprising 75 experts in rheumatology, nephrology, dermatology, obstetrics, radiology, and evidence-based medicine. Methodological support was provided by the Evidence-Based Medicine Center of Lanzhou University, the Lanzhou University GRADE (The Grading of Recommendations Assessment, Development and Evaluation) Center, and the Evidence-Based Evaluation and Guideline Innovation Unit of the Chinese Academy of Medical Sciences. All working group members completed conflict-of-interest disclosure forms, confirming no direct interest conflicts related to this guideline.

### Guideline Registration and Protocol Development

The guideline was registered at the International Practice Guidelines Registry Platform (http://www.guidelines-registry.org; registration number: PREPARE-2024CN1085). Its development adhered to the World Health Organization (WHO) Handbook for Guideline Development (2014),^[19]^ the Basic Methods and Procedures for Developing/Revising Clinical Practice Guidelines issued by the Chinese Medical Association (2016),^[20]^ and incorporated the Appraisal of Guidelines for Research and Evaluation II (AGREE II) instrument^[21]^ and the Reporting Items for Practice Guidelines in Healthcare (RIGHT) standards.^[22,23]^

### Guideline Users and Target Population

This guideline is intended for rheumatologists, nephrologists, dermatologists, obstetricians, clinical pharmacists, radiologists, and other healthcare professionals involved in SLE diagnosis and management. The recommendations apply to patients with confirmed SLE.

### Selection and Prioritization of Clinical Questions

Through systematic reviews of published SLE guidelines and systematic reviews, supplemented by interviews with rheumatology experts, the working group initially identified 30 clinical questions. An online survey assessed their importance, with two rounds of feedback from 83 participants. Twelve key clinical questions were ultimately selected for inclusion.

### Evidence Retrieval

For each clinical question and outcome, the PICO (Population, Intervention, Comparison, Outcome) framework guided literature searches in: (1) MEDLINE, Cochrane Library, Web of Science, Embase, CBM (China Biology Medicine), Wanfang, and CNKI (China National Knowledge Infrastructure, from the establishment of the database to October 2024), prioritizing systematic reviews, meta-analyses, and network meta-analyses, followed by randomized controlled trials (RCTs), cohort studies, case-control studies, case series, and epidemiological surveys; (2) Official websites of the National Institute for Health and Care Excellence (NICE), the National Guideline Clearinghouse (NGC), the Scottish Intercollegiate Guidelines Network (SIGN), ACR, EULAR, and APLAR for SLE-related studies and guidelines; (3) Supplementary searches in Epistemonikos, UpToDate, DynaMed, and Google Scholar.

### Evidence Evaluation and Grading

The evidence team assessed bias risk using: A Measurement Tool to Assess Systematic Reviews (AMSTAR) for systematic reviews, meta-analysis, and network meta-analysis;^[24]^ Cochrane Risk of Bias (ROB) tool for RCTs;^[25]^ Quality Assessment of Diagnostic Accuracy Studies (QUADAS-2) for diagnostic studies;^[26]^ Newcastle-Ottawa Scale (NOS) for observational studies.^[27]^ Two independent reviewers conducted evaluations, with discrepancies resolved through discussion or third-party consultation. Evidence quality and recommendation strength were graded using the GRADE (Grading of Recommendations Assessment, Development, and Evaluation) approach (Table 1).^[28, 29, 30, 31, 32]^

**Table 1:** Grading of Recommendations Assessment, Development, and Evaluation

GRADE Rating	Description
Quality of Evidence	
High (A)	High confidence: The estimated effect lies close to the true value
Medium (B)	Moderate confidence: The estimated effect is likely close to the true value, but may be substantially different
Low (C)	Low confidence: The estimated effect may be substantially different from the true value
Very Low (D)	Very low confidence: The estimated effect is likely substantially different from the true value
Strength of Recommendation	
Strong (1)	Clearly demonstrates that benefits outweigh harms or vice versa
Weak (2)	Uncertain balance of benefits and harms, or evidence shows comparable benefits and harms regardless of quality

### Recommendation Formulation

Based on the synthesized evidence (including domestic and international studies) provided by the evidence evaluation team, and after comprehensive consideration of Chinese patients’ preferences and values, intervention costs, and benefit-risk profiles, the expert panel drafted initial recommendations specific to China’s clinical practice. A Delphi survey was conducted on December 11th, 2024, which collected 30 feedback suggestions. Face-to-face consensus meetings held on November 22nd and December 19th, 2024, further refined the recommendations. Final consensus was achieved for all recommendations (defined as ≥75% expert agreement).

### Guideline Update

The working group will continuously monitor emerging evidence and the applicability of current recommendations. The recommendations of this guideline are planned to be updated within 3–5 years in accordance with the methodologies in International Guidelines.^[33]^

**Question 1: How to diagnose SLE?**

**Recommendation 1: The 2012 Systemic Lupus International Collaborating Clinics (SLICC) classification criteria or the 2019 EULAR/ACR SLE classification criteria are recommended for the diagnosis of patients with suspected SLE (1B). For patients with atypical clinical presentations or diagnostic difficulties, consultation with or referral to a rheumatology specialist is recommended (2C).**

To improve the sensitivity and specificity of SLE classification criteria, the 2019 EULAR/ACR criteria were developed. These include one entry criterion and 18 criteria across 10 domains. Each criterion must exclude causes such as infection, malignancy, or drug. Criteria do not have to occur simultaneously. Within each domain, only the highest weighted criterion is counted toward the total scores. Patients who meet the entry criterion and receive scores of 10 or more are classified as SLE.^[34]^ Validation studies showed that the 2019 EULAR/ACR, the 2012 SLICC, and the 1997 ACR criteria had sensitivities of 96%, 97%, and 83%, and specificities of 93%, 84%, and 93%, respectively, with the 2019 EULAR/ACR criteria demonstrating the optimal balance.^[34]^ However, in adult SLE patients, the 2012 SLICC criteria showed superior sensitivity (100%) and specificity (75%) compared with the 2019 EULAR/ACR criteria (93% sensitivity, 73% specificity) and the 1997 ACR criteria (83% sensitivity, 82% specificity).^[35]^ Thus, this guideline recommends using both the 2019 EULAR/ACR and the 2012 SLICC classification criteria for SLE diagnosis in China. To avoid missing diagnoses in persistently antinuclear (ANA)-negative individuals, the 2012 SLICC criteria should be applied concurrently.

SLE shares overlapping clinical and laboratory features with primary Sjögren’s syndrome, rheumatoid arthritis, and mixed connective tissue disease. Detailed clinical evaluation and autoantibody testing are critical for differentiation. The 2019 EULAR/ACR criteria significantly reduce misdiagnosis. A retrospective study (352 SLE patients *vs*. 385 disease controls) from China found both the 2012 SLICC and the 2019 EULAR/ACR criteria effective for early diagnosis, with the latter performing better.^[36]^ A cross-sectional study from another country revealed that only 23% of SLE cases diagnosed by primary care physicians met the 1997 ACR criteria (fulfilled 4 or more criteria), compared to 79% of cases diagnosed by rheumatologists.^[37]^ This highlights the importance of the involvement of rheumatology specialists in SLE diagnosis. A review published in *The Lancet* in 2024 emphasized the challenges primary care physicians face when diagnosing SLE and recommended early referral to rheumatologists.^[38]^

**Question 2: What are the treatment principles and goals for SLE?**

**Recommendation 2: The principles of SLE treatment emphasize early intervention and a Treat-to-Target (T2T) strategy (1C). Short-term goals include controlling disease activity and achieving clinical remission or at least low disease activity (1C). Long-term goals include maintaining remission, preventing flares, minimizing drug toxicity, reducing organ damage, improving quality of life, and lowering mortality (1C).**

The average delay in diagnosing SLE in China is 10.8 months, ^[39]^ which is associated with increased disease activity and organ damage.^[40]^ A meta-analysis confirmed that active disease worsens quality of life ^[41]^ and increases mortality,^[42]^ underscoring the need for early intervention.

The Treat-to-Target (T2T) strategy involves adjusting therapy timely to achieve defined, feasible, and clinically meaningful targets. The 2021 DORIS (Definitions Of Remission In SLE) defines clinical remission ^[43]^ (Table 2). If remission is unattainable, the Asia-Pacific Lupus Collaboration (APLC)-proposed Lupus Low Disease Activity State (LLDAS) serves as an alternative.^[44]^

**Table 2:** Definitions of SLE Clinical Remission and Low Disease Activity

Treatment Target	Clinical Remission^[43]^	Low Disease Activity^[44]^
Definition	• Clinical SLEDAI (cSLEDAI) = 0	• SLEDAI-2K ≤4
	• Physician Global Assessment (PGA) ≤0.5	• PGA ≤1
	• Permitted therapies: antimalarials (*e.g*., hydroxychloroquine), lowdose glucocorticoids (prednisone ≤5 mg/d or equivalents), and/or stable-dose immunosuppressants/biologics	• Permitted therapies: antimalarials, low-dose glucocorticoids (prednisone ≤7.5 mg/d or equivalents), and/or stable-dose immunosuppressants/biologics

A systematic review of 41 studies (17, 270 patients) found that 42.4%-88% of patients achieved remission within one year, and 21.1%-70% sustained remission for five years or longer. ^[45]^ Another systemic review indicated that remission (OR = 0.49–0.75) and LLDAS (OR = 0.19–0.88) significantly reduce the SLICC/ACR Damage Index (SDI), correlating with lower risks of organ damage, flares, mortality, and other adverse outcomes (such as death, severe infections, and hospitalization risks) compared to uncontrolled disease activity. ^[46]^ Even after achieving remission/LLDAS, close monitoring is critical for preventing disease flare and optimizing treatment. Maintaining these states for more than 3–6 months reduces the risks of flares and organ damage, with extended maintenance further improving outcomes. ^[47,48]^

**Question 3: How to assess SLE disease activity and organ damage?**

**Recommendation 3: It is recommended to use SLE disease activity index (SLEDAI**⁃**2000) score combined with the clinician’s comprehensive assessment to evaluate SLE disease activity (2C). Assessment of disease activity is recommended at least once a month for patients with active SLE and once every 3 to 6 months for patients with stable SLE (2D). Disease flares should be treated as active SLE (2D).**

Multiple tools are available for SLE activity assessment,^[49, 50, 51, 52, 53, 54, 55, 56, 57, 58, 59]^ all requiring integration of past medical history, physical examination, and laboratory tests. The physician’s personal preference and expertise, the cost of evaluation (whether a computer is needed and the expenses of examination), and the time required all influence the choice of evaluation tools.^[60]^ SLEDAI-2000 scores range from 0 to 105, while British Isles Lupus Assessment Group (BILAG)-2004 scores are divided into 5 categories: A, B, C, D and E. Compared with BILAG-2004, SLEDAI-2000 takes less time for assessment and is easier to use, which is preferred.^[61,62]^

Disease activity can be graded based on SLEDAI-2000. At present, there are mainly four criteria for grading disease activity,^[12,13,43,44]^ and the EULAR’s criteria are recommended, which define mild activity as SLEDAI-2000 ≤6, moderate activity as SLEDAI-2000 7–12, and severe activity as SLEDAI-2000 >12.^[12]^ Because a higher SLEDAI-2000 is associated with increased risks of organ damage (HR = 1.18, 95% CI: 1.02–1.37) and death (HR= 1.14, 95% CI: 1.02–1.22),^[63]^ regular monitoring of disease activity and organ damage is needed. Since evaluating disease activity based solely on SLEDAI-2000 and BILAG-2004 has certain limitations, it is also necessary to combine the Physician’s Global Assessment (PGA) with reference to the patient’s clinical manifestations to improve the accurance of the evaluation.

The Systemic Lupus Erythematosus Disease Activity Score (SLE-DAS) was developed by Jesus *et al* in 2019. The aim is to address the low sensitivity of SLEDAI-2000 in capturing changes in disease activity through a continuous scoring method, while maintaining high specificity and clinical usability.^[54]^ SLE-DAS includes 17 clinical and laboratory indicators, such as hemolytic anemia, cardiopulmonary manifestations, and gastrointestinal involvement, which are absent in SLEDAI-2000. SLE-DAS is calculated using a mathematical formula, with a strong correlation with PGA and SLEDAI-2000 (*r* = 0.875 and *r* = 0.943, respectively). In validation study, SLE-DAS was significantly more sensitive to disease improvement and deterioration than SLEDAI-2000, with a greater ability to predict damage accumulation. The calculation of SLE-DAS is relatively complex and relies on formula calculations. According to the score, the disease activity can be divided into mild activity (SLE-DAS ≤2.08), moderate activity (2.08<SLE-DAS≤7.64), and severe activity (SLE-DAS >7.64).^[64]^

The Systemic Lupus International Collaborating Clinics (SLICC) Damage Index (SDI) is the only internationally recognized and validated assessment standard for SLE organ damage, which scores 12 organ systems independently and is an effective tool for evaluating organ damage in clinical practice. SDI provides a basis for better predicting the prognosis of SLE patients.^[65]^

There is a lack of evidence regarding the frequency of monitoring disease activity in SLE patients. The UK SLE guidelines recommend evaluating disease activity at least once every 1–3 months in patients with active disease, while the Spanish SLE guidelines recommend evaluations at least once every 3–4 months in the first year. For SLE patients with stable or low disease activity, both the UK and Spanish SLE guidelines recommend an evaluation every 6–12 months.^[13,66]^ Based on the above international guideline recommendations, this guideline recommends assessing disease activity at least once a month for patients with active SLE (83.33% consensus), and once every 3–6 months for patients with stable SLE (94.44% consensus).

In addition, the frequency of clinical monitoring should be adjusted according to the progression of the disease and the intensity of treatment. SLE flares should be treated as active disease, and the disease activity evaluation should be performed at least once a month until SLE is stable.

**Question 4: How to use glucocorticoids in the treatment of SLE?**

**Recommendation 4: Glucocorticoid is the foundational medication for the treatment of SLE (1A). An individualized glucocorticoid regimen should be formulated based on disease activity, the involved organs, and its severity, at the lowest effective dose (1B). For mild SLE, low-dose glucocorticoids (≤10 mg/d prednisone or equivalent) should be considered if hydroxychloroquine or nonsteroidal anti-inflammatory drugs (NSAIDs) are ineffective. For moderate SLE, glucocorticoids (0.5–1 mg/kg/d prednisone or equivalent) combined with immunosuppressants are recommended (2C). For severe SLE, high-dose glucocorticoids (≥1 mg/kg/d prednisone or equivalent) combined with immunosuppressants should be used (2C). For life-threatening organ involvement, pulse glucocorticoid therapy combined with immunosuppressants is recommended (1B). Clinicians should closely monitor disease activity and adjust glucocorticoid doses accordingly. For patients with long-term stable disease, the maintenance dose should be ≤5 mg/d prednisone or equivalent, with an attempt to discontinue if possible (1B).**

Glucocorticoids play a crucial role in SLE treatment and are the most commonly used agents for induction therapy, widely recommended in both domestic and international guidelines.^[12,13,67, 68, 69]^ Glucocorticoid regimens should be individualized based on disease activity, organ involvement, and severity, with dose adjustments guided by treatment response, duration, and adverse effects.

For mild SLE, low-dose glucocorticoids (≤10 mg/d prednisone or equivalent) may be considered if hydroxychloroquine or NSAIDs fail to control disease activity.^[13,16,66,68]^ For patients with moderately active SLE, medium-dose glucocorticoids (0.5–1 mg/kg/d prednisone or equivalent) are recommended. If disease activity is not rapidly controlled with medium-dose glucocorticoids, combination therapy with immunosuppressants is advised to reduce cumulative steroid exposure and minimize the risk of long-term adverse effects.^[13,16]^ For severe SLE, high-dose glucocorticoids (1 mg/kg/d prednisone or equivalent) combined with immunosuppressants are recommended, with dose adjustments after stabilization. Pulse glucocorticoid therapy may be used if necessary.^[12,66,68]^ For life-threatening organ involvement, pulse glucocorticoid therapy (intravenous methylprednisolone 250–1000 mg/d for 3 days, repeatable at 5–30-day intervals if needed) combined with immunosuppressants is recommended. Following pulse therapy, oral prednisone (0.5–1 mg/kg/d or equivalent) should be given for 4–8 weeks, with dose modified according to patient response. Compared with high-dose oral glucocorticoids, pulse therapy achieves faster disease control, allows quicker tapering, and does not significantly increase the risk of adverse effects.

Dosage adjustment and tapering of glucocorticoids should be based on disease activity and the occurrence of steroid-related adverse effects. The tapering process must be gradual and carefully monitored to avoid abrupt withdrawal. For stable patients, early dose reduction is recommended, with the goal of maintaining ≤5 mg/d prednisone or equivalent, and discontinuation if feasible. Real-world studies show that patients maintained on <5 mg/d prednisone or equivalent have less damage accumulation, while those never tapered below 5 mg/d face higher risk of steroid-related adverse effects.^[69]^ A global multicenter prospective observational cohort study demonstrated that in SLE patients meeting the criteria for modified serologic activity (elevated anti-dsDNA and/or hypocomplementemia) with clinically quiescent disease (SACQ), glucocorticoid tapering did not increase the risk of disease flares and exhibited a protective effect against damage accumulation.^[70]^ A meta-analysis indicated that glucocorticoid discontinuation may slightly increases the risk of flares (mainly mild flares) but reduces the incidence of SDI progression.^[71]^

Glucocorticoid-related adverse effects occur in >30% of patients. The most common short-term adverse reactions are gastrointestinal disturbances, excitation, palpitations, and insomnia, while long-term adverse reactions include osteoporosis, osteonecrosis of the femoral head, secondary infection, glaucoma, and cataracts.^[72]^ The occurrence of adverse effects from glucocorticoid therapy is positively correlated with dosage. Therefore, clinicians should aim to prescribe the minimal effective dose required for disease control, concurrently avoiding potential risks stemming from inadequate dosage or inappropriate administration.

**Question 5: How to use hydroxychloroquine in the treatment of SLE?**

**Recommendation 5: Long-term hydroxychloroquine is recommended as the mainstay of treatment for patients with SLE (1A). For patients taking hydroxychloroquine, ocular risk assessment is recommended. For high-risk patients, an annual ophthalmologic examination is recommended. Low-risk patients are recommended to undergo an ophthalmologic examination annually from the fifth year of medication (2C).**

Hydroxychloroquine (HCQ) is a cornerstone therapy for patients with SLE. Long-term use of HCQ reduces disease activity, decreases the risk of organ damage and thrombosis, and improves lipid profiles and survival.^[73, 74, 75]^ The recommended dose is 5 mg/kg/d (real body weight), not exceeding 400 mg/d. The dosage may be individualized based on the patient’s risk of flares and retinal toxicity. A recent follow-up study of 342 SLE patients found that using HCQ at ≤5 mg/kg/d, compared to >5 mg/kg/d, significantly increased the risk of disease relapse (OR = 1.98, 95% CI: 1.03–3.79).^[76]^ An additional study of the SLICC cohort showed that both dose reduction (HR = 1.20, 95% CI: 1.04–1.38) and discontinuation (HR = 1.56, 95% CI: 1.31–1.86) of HCQ significantly elevated the risk of flares. ^[77]^ High-risk factors for HCQ-related retinal toxicity include long-term and/or high-dose ( >5 mg/kg/d) use, hepatic or renal impairment, concurrent use of tamoxifen, a history of retinal or macular disease, and age over 45 years.^[78, 79, 80]^ Among individuals administered HCQ at ≤5 mg/kg/d, the risk of toxicity is under 1% over 5 years and under 2% over 10 years, but it rises to almost 20% after 20 years. However, for patients who remain toxicity-free after 20 years of use, the future probability of developing toxicity is only 4%.^[81]^ Therefore, for patients without high-risk factors, a baseline ophthalmologic examination is recommended, followed by annual screenings (such as visual field testing and spectral-domain optical coherence tomography [SD-OCT]) starting after five years of treatment to monitor for drug-induced retinal toxicity.^[81,82]^ In contrast, patients at high risk for retinal toxicity should undergo annual ophthalmologic examinations both before and during HCQ treatment to enable early detection and intervention.

Contraindications for HCQ include hypersensitivity to HCQ or its derivatives and a history of retinopathy caused by similar drugs. Recent studies suggest that HCQ may be associated with QT interval prolongation^[83, 84, 85]^ and cardiac conduction blocks.^[86]^ Therefore, patients with preexisting cardiac conditions or those taking concomitant medications (such as Class I and III antiarrhythmic agents, tricyclic antidepressants, antipsychotics, and certain anti-infective drugs such as macrolides and fluoroquinolones) should be monitored for arrhythmic risk.^[85]^ Additionally, case reports have suggested a potential association between long-term HCQ use and cardiomyopathy,^[86, 87, 88, 89]^ though the exact causality and specific risk factors require further investigation.

**Question 6: How to use immunosuppressants and biologics in the treatment of SLE?**

**Question 6.1: How to use immunosuppressants in the treatment of SLE?**

**Recommendation 6.1: Immunosuppressants are recommended for patients who do not respond well to glucocorticoids in combination with HCQ or who are unable to taper glucocorticoids to a maintenance dose (≤5 mg/d of prednisone or an equivalent dose of other glucocorticoids) (1B). Immunosuppressants are recommended at the time of initial treatment for patient with organ involvement (2B).**

The use of immunosuppressants reduces the cumulative dose of glucocorticoids and prevents disease flares.^[90]^ In patients with refractory (poor response to conventional therapy) or flared SLE, immunosuppressants can reduce glucocorticoids dosage, control disease activity and improve clinical remission rates.^[91,92]^ For patients with severe SLE with major organ involvement, high-dose cyclophosphamide (CTX) pulse therapy should be considered.^[12]^

For SLE patients with active skin lesions who show inadequate response to local treatments (such as topical glucocorticoids or tacrolimus ointment) and hydroxychloroquine, oral glucocorticoids combined with immunosuppressants can be added. Methotrexate (MTX) and mycophenolate mofetil (MMF) have comparable efficacy,^[93]^ and thalidomide and lenalidomide may be considered for patients who do not respond to other treatments.^[94,95]^ For lupus nephritis (LN) patients undergoing initial treatment (induction phase), compared to glucocorticoid monotherapy, the combination of immunosuppressants significantly improves clinical remission rates and reduces treatment failure. Therefore, immunosuppressants or multitarget therapy should be considered from the beginning of treatment.^[96, 97, 98]^ For SLE patients with organ involvement, the choice of immunosuppressants should be based on a comprehensive assessment of clinical manifestations, fertility issues, drug safety, and cost (Table 3).

**Table 3:** Indications, Advantages and Common and Significant Adverse Reactions of Different Immunosuppressants

Immunosuppressants	Main Applicable Populations	Advantages	Common Adverse Reactions
Mycophenolate Mofetil (MMF)	Moderate to severe SLE^[91]^	In patients with moderate to severe lupus nephritis, mycophenolate mofetil is an effective treatment in both the induction and maintenance phases, and in reducing the flare rate.^[109]^ It can also be used for the induction and maintenance treatment of mild to moderate neuropsychiatric lupus.^[110]^ For patients with mild to moderate SLE without organ involvement, MMF can help reduce the risk of severe flares and the occurrence of lupus nephritis.^[111]^	The most common adverse reactions are gastrointestinal discomfort. Some patients develop infections, myelosuppression and liver damage.^[112]^ Due to the teratogenicity, pregnancy can only be attempted at least 6 weeks after discontinuation.^[113]^
Cyclophosphamide (CTX)	Moderate to severe lupus nephritis, neuropsychiatric lupus and SLE with immune thrombocytopenia^[90,97,114]^	It is an effective treatment in the induction and maintenance phases for patients with moderate to severe lupus nephritis, and is an effective immunosuppressive agent for the treatment of neuropsychiatric lupus and hematological disorders.^[97,114]^	The common adverse reactions include gastrointestinal discomfort, nausea, and vomiting. Liver damage and myelosuppression are the main adverse reactions. It has well-documented reproductive toxicity and may increase the risk of malignancy.^[90,115,116]^ Glutathione S-transferase Pi 1 (GSTP1) gene polymorphism (rs1695, 313A > G) may be useful in predicting adverse reactions.^[117]^
Leflunomide (LEF)	Proliferative lupus nephritis^[118,119]^	It is effective and well tolerated in some patients with proliferative lupus nephritis.^[119]^ It can be used for the maintenance treatment of lupus nephritis.^[120]^	LEF can cause liver damage, hypertension, leukopenia, infections, and other complications. Due to its teratogenicity, it is recommended to discontinue LEF for 24 months before attempting pregnancy. Alternatively, cholestyramine (8 g orally, three times daily for 11 days) can be used for drug elimination. If two blood drug concentration tests, taken two weeks apart, both show levels <0.02 mg/L, LEF can be considered cleared, and pregnancy may be attempted.^[121]^
Methotrexate (MTX)	Mild to moderate SLE without renal involvement^[90]^	It is effective in improving skin, arthritis and overall condition in patients with SLE.^[90,122,123]^	The most common adverse reactions are gastrointestinal discomfort, such as nausea and vomiting. Hematological adverse reactions such as anemia, leukopenia and liver damage are also common.^[123]^
Tacrolimus (TAC)	Proliferative lupus nephritis, refractory lupus nephritis and SLE with immune thrombocytopenia^[109]^	It is an effective treatment in the induction and maintenance phases for patients with lupus nephritis. It can also reduce flares.^[109,127]^ It can be used for the treatment of refractory lupus nephritis, especially in those with proteinuria.^[124,125]^ Compared with other immunosuppressants or glucocorticoids, it has a low risk of causing serious infections.^[128]^ It can be used during pregnancy.^[129,130]^	The common adverse reactions include gastrointestinal discomfort, and some patients may experience kidney and liver damage. In patients with impaired liver function, the TAC dosage should be reduced. During treatment, renal toxicity, blood glucose, serum uric acid, and blood pressure should be monitored.^[127,131]^
Cyclosporine (CsA)	Lupus nephritis and immune thrombocytopenia	Cyclosporine in combination with other immunosuppressants may be used to treat lupus nephritis especially type V that does not respond to standard therapy.^[135]^ It is effective in some patients with hematological involvement,^[132,134]^ and can be used during pregnancy.^[129,130]^	The common adverse reactions include kidney damage, liver damage, increased blood pressure, gastrointestinal discomfort, and infections.^[134]^
Azathioprine (AZA)	Moderate SLE^[113]^	It can be used as the maintenance treatment for SLE. It has a low risk of severe infection. It is safe during pregnancy.^[113]^	The common adverse reactions are myelosuppression and liver damage.^[113]^ Testing for thiopurine methyltransferase activity is necessary.
Sirolimus	Moderate SLE^[101]^	It can be added for SLE patients with inadequate response to standard therapy. It can improve arthritis, rashes, and thrombocytopenia.^[100,101]^ It can be used as the maintenance therapy for lupus nephritis.^[104,105]^	The common adverse reactions are upper respiratory tract infections, gastrointestinal reactions, menstrual irregularities, oral ulcers, cytopenia, rash, and lipid abnormalities.^[101,103]^

Sirolimus, also known as rapamycin, was first reported in 2006 as a potential treatment for active SLE patients who have inadequate responses to conventional immunosuppressive therapy. Sirolimus treatment at a dose of 2 mg/d decreases disease activity and allows glucocorticoid tapering.^[99]^ A prospective, single-arm, open-label, single-center clinical trial in 2018 showed that 55% of patients experienced a significant reduction in disease activity (SLEDAI-2000 and BILAG-2004), and 66% had a response according to the SLE Responder Index (SRI) after one year of treatment. ^[100]^

A prospective real-world study conducted in China included 49 SLE patients with persistent disease activity despite standard immunosuppressive therapy. Following oral sirolimus treatment, SLEDAI-2000 scores significantly decreased, prednisone dosage was reduced, and serological markers (complement levels and anti-dsDNA antibodies) improved. Specifically, among LN patients, 41.2% achieved renal remission.^[101]^ A real-world cohort study from CSTAR demonstrated that sirolimus had comparable eficacy to tacrolimus in patients with active SLE.^[102]^ A meta-analysis further confirmed that sirolimus treatment leads to reductions in SLEDAI-2000, BILAG-2004 scores, and prednisone dosage in patients with active SLE.^[103]^ Additionally, small-scale non-randomized controlled studies suggested that sirolimus may be used as the maintenance treatment for LN,^[104,105]^ refractory SLE with immune thrombocytopenia,^[106,107]^ and SLE with concomitant antiphospholipid syndrome.^[108]^

**Question 6.2: How to use biologics in the treatment of SLE?**

**Recommendation 6.2: For patients who fail to achieve treatment targets or experience flares despite receiving glucocorticoid and conventional immunosuppressant therapy, biologic agents may be considered (1B).**

In recent years, an increasing number of biologics have been used to treat SLE, some of which have been approved for SLE treatment in China. Currently, biologics for SLE include agents primarily targeting B cells, such as belimumab, telitacicept, rituximab, and obinutuzumab, as well as the type I interferon receptor antagonist anifrolumab, and the C5 complement inhibitor eculizumab (Table 4).

**Table 4:** Indications, Advantages, and Major Adverse Reactions of Different Biologic Agents

Biologic Agent	Target	FDA or CFDA Approval Status	Usage Recommendations
Belimumab	Soluble B lymphocyte stimulator (BLyS)	Yes (FDA and CFDA approval)	It is the first biological agent approved for SLE treatment. It can significantly increase SRI-4 response rates, reduce disease activity and flares, and help reduce glucocorticoid and immunosuppressant dosage. It is recommended for combination therapy in patients with active SLE or LN.
Telitacicept	BLyS and a proliferation-inducing ligand (APRIL)	Yes (CFDA approval)	It can significantly improve SRI-4 response rates, ameliorate SLE disease activity and proteinuria levels, and facilitate glucocorticoid and immunosuppressant tapering.
Rituximab	CD20	No	It is effective for some patients with severe refractory SLE and LN, and can improve symptoms of neuropsychiatric lupus and hematologic involvement. It is recommended for patients with poor response to initial treatment.
Obinutuzumab	CD20	No	As a fully humanized CD20-targeted antibody, it is effective for LN patients, LN patients unresponsive to rituximab and SLE patients without renal involvement. It can improve renal remission rates and sustain renal responses.
Anifrolumab	type I interferon receptor	Yes (FDA approval)	It can be used to treat moderate-to-severe SLE, improving SRI-4 and BICLA response rates, enhance complete renal response in active class III/IV LN, and reducing glucocorticoid dosage and flare rates. It is recommended for patients who have difficulty tapering glucocorticoids.
Eculizumab	C5	No	It has been reported to be effective in some cases of refractory LN, thrombotic microangiopathy secondary to SLE, and antiphospholipid syndrome secondary to SLE

Belimumab is a human immunoglobulin G1 monoclonal antibody that inhibits the binding of soluble B lymphocyte stimulator (BLyS) to B cells. By blocking the binding of soluble BLyS to its receptors on B cells, it inhibits B-cell proliferation and is the first biologic approved globally for SLE treatment. The phase III trials BLISS-52, BLISS-76, and a study primarily involving Chinese patients in Northeast Asia, demonstrated that belimumab significantly increased the SLE Responder Index 4 (SRI-4) response rate, reduced SLE disease activity and risk of severe flares, and exhibited a favorable overall safety profile.^[136, 137, 138]^ Another international multicenter phase III trial (BLISS-LN) showed that adding belimumab in conjunction with standard care improved renal response rates and significantly reduced the probability of kidney-related events or death ^[139]^ in patients with active LN. A post hoc analysis of BLISS-LN revealed that belimumab in conjunction with standard therapy can reduce the risks of renal flare and decrease in estimated glomerular filtration rate (eGFR) in LN patients.^[140]^ Real-world studies have also confirmed that belimumab improved various SLE clinical manifestations, including renal, cutaneous, and joint involvement, and was associated with glucocorticoid dose reductions.^[141, 142, 143]^ The 2023 EULAR guidelines recommend belimumab for SLE patients who do not respond to hydroxychloroquine (either alone or combined with glucocorticoids), or who are unable to reduce glucocorticoids to maintenance doses, or who have active proliferative LN.^[12]^ The 2024 KDIGO guidelines recommend that patients with repeated kidney flares or at high-risk for progression to kidney failure due to severe chronic kidney disease should receive a triple immunosuppressive regimen of glucocorticoids combined with mycophenolate mofetil and belimumab, or glucocorticoids combined with reduced-dose cyclophosphamide and belimumab, which may also be continued as maintenance therapy.^[144]^ Additionally, studies have shown that belimumab is effective in treating active cutaneous manifestations.^[12]^

Telitacicept is a dual-target biologic against BLyS and a proliferation-inducing ligand (APRIL) that was independently developed and manufactured in China. Telitacicept received approval from the National Medical Products Administration in March 2021 for the treatment of SLE. Phase IIb trials showed telitacicept in conjunction with standard therapy significantly improved SRI-4 response, reduced disease activity and glucocorticoids dose.^[145]^ Phase III data demonstrated a SRI-4 response of 82.6% at 52 weeks.^[146]^ Real-world studies reported a SRI-4 response of 80% by week 45, with LN patients showing significantly reduced proteinuria and glucocorticoid dosage reduction.^[147]^ Retrospective analyses confirmed that telitacicept decreased disease activity, increased LLDAS achievement, reduced glucocorticoid dosage, and improved both renal and hematologic parameters.^[148,149]^

Rituximab, an anti-CD20 monoclonal antibody, showed efficacy in refractory SLE despite failing to meet its primary efficacy endpoint in EXPLORER and LUNAR trials.^[150,151]^ Observational studies and meta-analyses supported its efficacy in severe refractory SLE.^[152,153]^ EULAR 2023 guidelines recommend rituximab for refractory LN,^[12]^ and KDIGO 2024 guidelines recommend it for active LN unresponsive to initial therapy.^[144]^ The 2021 Chinese LN guidelines recommend it for proliferative LN refractory to conventional immunosuppressants.^[154]^ Additionally, rituximab can rapidly alleviate central nervous system symptoms in patients with refractory neuropsychiatric lupus, including cognitive impairment, neurological symptoms, and epilepsy. It has also been shown to improve corresponding imaging findings.^[155]^ A systematic review study showed that rituximab treatment for neuropsychiatric lupus achieved response rates as high as 73%-100%.^[110]^ A multicenter retrospective cohort study demonstrated that rituximab yielded an initial treatment response rate of 86% in SLE patients with hematologic involvement, with specific response rates of 91% for immune thrombocytopenia, 87.5% for autoimmune hemolytic anemia, and 60% for Evans syndrome.^[156]^ A meta-analysis confirmed an improved complete remission rate in SLE patients with thrombocytopenia without increased infection risk.^[157]^ Therefore, 2023 EULAR guidelines recommend to consider rituximab for acute treatment of severe autoimmune thrombocytopenia.^[12]^

Obinutuzumab is a fully humanized type II anti-CD20 monoclonal antibody that exhibits stronger B-cell cytotoxic effects compared to rituximab. The phase II NOBILITY trial investigating obinutuzumab in active LN demonstrated a significantly higher complete renal response rate at week 52 compared to placebo, with sustained renal improvement persisting through week 104.^[158]^ The phase 3 REGENCY trial further confirmed a superior complete renal response rate at week 76 for obinutuzumab combined with standard therapy *versus* placebo, particularly in patients with higher baseline disease activity (UPCR ≥3 g/g, hypocomplementemia, high-titer anti-dsDNA antibodies, class IV LN, or mixed class V LN).^[159]^ Obinutuzumab also demonstrated efficacy in rituximab-refractory LN and non-renal SLE, with additional benefits in reducing overall disease activity.^[160]^

Anifrolumab, a type I interferon receptor antagonist, has been approved by the U.S. Food and Drug Administration for moderate-to-severe SLE. The phase 2b MUSE trial showed that anifrolumab could significantly increase the proportion of patients achieving SRI-4 response, reduce oral glucocorticoid dose, improve the Composite Lupus Assessment (BICLA) response based on BILAG, and alleviate joint swelling or tenderness and rash compared to placebo. Notably, the anifrolumab group exhibited significantly lower SLE flare rates.^[161]^ Post-hoc analysis of MUSE revealed patients of anifrolumab group achieved LLDAS faster and maintained LLDAS longer than those of placebo group.^[162]^ Although the phase 3 TULIP-1 trial did not meet its primary endpoint of SRI-4 response, anifrolumab significantly outperformed placebo in glucocorticoid tapering, CLASI score improvement, joint symptom relief, and BICLA response rates.^[163]^ TULIP-2 confirmed greater BICLA response rates in anifrolumab group, with more patients achieving prednisone ≤7.5 mg/d and ≥50% CLASI score reduction *versus* placebo.^[164]^ The TULIP-LN trial demonstrated enhanced complete renal responses in active class III/IV LN patients receiving intensive anifrolumab therapy.^[165]^ Based on the aforementioned research findings, the 2023 EULAR guidelines recommend adding anifrolumab for SLE patients who are unresponsive to hydroxychloroquine (either monotherapy or combined with glucocorticoids) or who are unable to taper glucocorticoids to maintenance doses.^[12]^

Eculizumab, a recombinant humanized monoclonal antibody selectively inhibiting complement C5, is indicated for paroxysmal nocturnal hemoglobinuria (PNH) and atypical hemolytic uremic syndrome (aHUS) in adults and children. Case reports suggested its potential efficacy in refractory LN, thrombotic microangiopathy secondary to SLE, and antiphospholipid syndrome secondary to SLE.^[166, 167, 168, 169, 170, 171]^ A systematic review reported a 93% response rate in LN-associated thrombotic microangiopathy patients treated with eculizumab.^[172]^

**Question 7: How to treat SLE with organ and systemic involvement?**

**Question 7.1: How to treat lupus nephritis?**

**Recommendation 7.1: For Class I and Class II LN with low-grade proteinuria that below the nephrotic range, treatment should be based on extrarenal manifestations (2C). For Class II LN with nephrotic range proteinuria, glucocorticoids and/or conventional immunosuppressants are recommended (2B). For active Class III or IV LN, with or without class V, induction therapy with glucocorticoids plus any one of the following is recommended:** ① **mycophenolate mofetil (MMF)(1B) or intravenous cyclophosphamide (IV-CTX)(1B), which may be combined with belimumab (1B); or** ② **calcineurin inhibitors (CNI) (1C); or** ③ **MMF and CNI (1C). As for maintenance therapy, MMF or azathioprine (AZA) is recommended (1B). If MMF or AZA is contraindicated or not tolerated, CNI or leflunomide may be considered (1C). For pure Class V LN, induction therapy should be stratified based on urinary protein levels (1B). For LN patients with persistent disease activity or inadequate response to initial standard-of-care therapy, addition of rituximab (RTX) may be considered (1B).**

The treatment principles and goals for LN are generally consistent with those for SLE, and renal pathology type and disease activity are fundamental to formulate LN treatment plans. The LN pathological classification is primarily based on the 2003 International Society of Nephrology/Renal Pathology Society (ISN/RPS) recommendations^[173]^ and the 2018 ISN/RPS revisions to LN pathological classification and the National Institutes of Health (NIH) activity/chronicity index scoring system.^[174]^

In current initial treatment regimens for LN, glucocorticoids are universally used. For patients with proliferative LN (with or without class V), it is recommended to start with intravenous methylprednisolone at a dose of 250–500 mg/d for 1–3 days, followed by oral prednisone ≤0.5 mg/kg/d.^[12,144]^ In cases with severe active lesions accompanied by acute kidney injury, the methylprednisolone pulse dose may be increased to 500–1000 mg/d, or the treatment duration may be extended. For class II and class V LN, oral glucocorticoid therapy is typically used, with the dose adjusted based on the severity of proteinuria, renal function, and extrarenal manifestations.

For Class I LN and Class II LN with proteinuria that below the nephrotic range, since renal damage is mild, treatment should be based on extrarenal manifestations of SLE.

For Class II LN with nephrotic range proteinuria, lupus podocytopathy should be considered. For lupus podocytopathy with mild glomerular lesions or mesangial proliferation, glucocorticoid monotherapy and glucocorticoid combined with immunosuppressant therapy have similar remission rates. For cases with focal segmental glomerulosclerosis (FSGS) lesions, the complete remission rate with glucocorticoid monotherapy is only 16.7%, while glucocorticoids combined with conventional immunosuppressants can increase the remission rate to 33.3%. Since the flare rate with glucocorticoid monotherapy is significantly higher than that with combination therapy (89.5% *vs*. 35.7%, *P* < 0.001),^[175]^ glucocorticoids combined with conventional immunosuppressants are recommended.

Class III/IV LN, with or without Class V LN, is a progressive disease requiring prompt and effective treatment to suppress renal immune-inflammatory lesions. A meta-analysis showed that for the induction therapy of proliferative LN, MMF and IV-CTX had similar complete remission rates (OR = 1.44, 95% CI: 1.00–2.06), as did CNI and IV-CTX (OR = 1.74, 95% CI: 1.09–2.79). As for the maintenance therapy, MMF had a lower risk of flares than AZA (OR = 0.53, 95% CI: 0.31–0.90), while CNI (OR = 0.64, 95% CI: 0.22–1.88) and IV-CTX (OR = 1.68, 95% CI: 0.51–5.51) showed no statistical difference in flare risk compared to AZA.^[97]^ A Chinese multicenter prospective open-label study included 270 LN patients who achieved remission with the induction therapy with glucocorticoids plus IV-CTX. These patients were randomly assigned to receive maintenance therapy with glucocorticoids plus either leflunomide (LEF) at a does of 20 mg/d or AZA at a target dose of 100 mg/d. At 36 months, there was no significant difference in rates of flares (15.7% *vs*. 17.8%) or adverse effects between the two groups.^[120]^

The triple immunosuppressive regimen of glucocorticoids, MMF and CNI is particularly suitable for LN patients with extensive podocyte injury causing massive proteinuria. The multi-target regimen including fixed-dose tacrolimus (TAC), reduced-dose MMF, and glucocorticoids significantly reduces proteinuria and maintains stable renal function. A multicenter RCT of 368 LN patients with active LN and baseline serum creatinine (SCr) ≤3 mg/dL showed that multi-target regimen had significantly higher cumulative complete response rates than IV-CTX (46% *vs*. 26%, *P* < 0.001) at 24 weeks, especially for Class IV+V LN (45% *vs*. 27%), with similar adverse event rates.^[176]^ Another study comparing multi-target maintenance therapy after multi-target induction therapy *versus* AZA maintenance therapy after IV-CTX induction therapy found no significant difference in renal flare rates (5.47% *vs*. 7.62%, *P* = 0.74) at 24-month, but AZA maintenance therapy has a significantly higher rate of adverse events (44.4% *vs*. 16.4%, *P* < 0.01).^[177]^ A meta-analysis of 8 trials (801 LN patients) showed multi-target induction therapy had superior complete response rates *versus* IV-CTX (RR [risk ratio] = 1.94, 95% CI: 1.61–2.33; *P* < 0.00001), particularly for Class IV (RR = 1.52), Class V (RR = 4.24) and Class IV+V LN (RR = 2.29). Multi-target therapy has lower rates of gastrointestinal symptoms, liver dysfunction, leukopenia and menstrual irregularities, but it increases the risk of new-onset hypertension. Notably, CNI should be used cautiously in patients with severe chronic kidney damage.^[178]^

The BLISS-LN study enrolled 448 LN patients who were randomly assigned to receive either belimumab or placebo in combination with standard therapy (MMF or CTX followed by AZA). At week 104, the belimumab treatment group showed significantly higher rates of primary renal response (43% *vs*. 32%; OR = 1.6; 95% CI: 1.0–2.3; *P* = 0.03) and complete renal response (30% *vs*. 20%; OR = 1.7; 95% CI: 1.1–2.7; *P* = 0.02) compared with the placebo group.^[139]^ In the East Asian subgroup of 142 LN patients, belimumab showed higher rates of primary renal response (53% *vs*. 37%; OR = 1.76; 95% CI: 0.88–3.51) and complete renal response rate (35% *vs*. 25%; OR = 1.73; 95% CI: 0.80–3.74) at 104 weeks, with significantly better primary response rate at 52 weeks (belimumab-to-placebo 62% to 37%; OR = 2.74; 95% CI: 1.33–5.64).^[179]^ A post-hoc analysis found that belimumab improved outcomes in both newly-diagnosed and flared LN,^[180]^ significantly reducing renal-related events, death and LN flare risk, especially in patients with proliferative LN with UPCR <3 g/g.^[140]^ For patients with class III/IV + V LN who respond to belimumab during the induction therapy phase, combination treatment with belimumab may be continued during the maintenance phase.^[144]^

For Class V LN, also referred as membranous LN, the initial treatment depends on the severity of proteinuria. For patients with proteinuria <1 g/24 h, the prognosis is generally favorable, and no specific immunosuppressive therapy targeting renal involvement is required. In these cases, treatment should be formulated based on extrarenal manifestations of SLE. General renal protective measures including renin-angiotensin-aldosterone system inhibitors (RAASi) and SGLT2 inhibitors are also recommended.^[181]^ Persistent high-grade proteinuria represents a higher risk for developing end-stage kidney disease (ESKD), cardiovascular events, and thrombotic complications in Class V LN, necessitating conventional immunosuppressive therapy to control proteinuria. Therefore, for patients with 24-hour urinary protein levels of 1.0–3.5 g, treatment with glucocorticoids combined with conventional immunosuppressants is recommended, while for those with 24-hour urinary protein levels >3.5 g/24 h, a regimen combining glucocorticoids with MMF plus CNI is preferred as the first-line therapy. During the maintenance phase, options include MMF, CNI, CNI combined with MMF, AZA, and tripterygium glycosides. A subgroup analysis of a Chinese multi-center study demonstrated that multi-target therapy showed significantly higher complete remission rates compared to IV-CTX (33% *vs*. 8%, *P* = 0.01) in Class V LN, with no significant difference in adverse event rates.^[176]^

RTX effectively depletes B cells and controls disease activity, serving as an important treatment option for refractory LN. A meta-analysis incorporating 31 studies involving 1, 112 SLE/LN patients showed that RTX combination therapy achieved overall, complete, and partial remission rates of 72%, 46%, and 32% respectively, and also reduced disease activity scores and glucocorticoid dosage.^[153]^ Obinutuzumab depletes B cells more effectively, significantly improves complete renal response rates, and remains effective even in LN patients with secondary non-response to rituximab.^[159,160]^

In addition to optimizing immunosuppressive therapy, the management of LN should also address non-immune factors contributing to disease progression. These include a low-salt diet (sodium intake <5 g/d), avoidance of high-protein intake, smoking cessation, moderate exercise, weight reduction (BMI, body mass index ≤25 kg/m^2^), preferential use of RAASi to achieve target blood pressure (<120/70 mmHg), reduction of proteinuria, and delay of chronic kidney disease progression.^[182]^

**Question 7.2 How to treat severe thrombocytopenia in SLE?**

**Recommendation 7.2: The primary treatment goal for SLE-associated immune thrombocytopenia is to minimize bleeding risk. In the acute phase, high-dose glucocorticoids (including intravenous methylprednisolone pulses) or intravenous immunoglobulin (IVIG) may be considered (1A). For patients with inadequate response, glucocorticoids combined with rituximab or conventional immunosuppressants are recommended (2B). Thrombopoietin receptor agonists (TPO-RAs) can be used for life-threatening bleeding events or emergency surgery (1A).**

Immune-mediated thrombocytopenia is a common hematologic manifestation of SLE, defined as a platelet count < 100×10^9^/L on two consecutive tests.^[34]^ However, alternative etiologies must be excluded, including viral infections (*e.g*., Epstein-Barr virus [EBV], cytomegalovirus [CMV]), thrombotic events, hypersplenism, drug-induced thrombocytopenia (*e.g*., heparin), and thrombotic microangiopathy.

Disease severity should be assessed based on platelet count and bleeding symptoms.^[183,184]^ The therapeutic objective is to maintain a safe platelet count to prevent bleeding, rather than normalize platelet counts. Typically, when the platelet count is greater than 50×10^9^/L, the bleeding risk is relatively low. Active treatment is warranted if the platelet count is ≤ 30×10^9^/L and/or the bleeding score is ≥2 points. For severe thrombocytopenia with a platelet count <10×10^9^/L or a bleeding score >5 points, the platelet count should be rapidly increased to a safe level.^[185]^ Currently, high-quality evidence and guidelines/expert consensus for SLE-associated thrombocytopenia are limited. Management strategies are largely extrapolated from primary immune thrombocytopenia (ITP) guidelines.

A retrospective cohort study involving 18 antinuclear antibody-positive ITP patients showed that glucocorticoid mono-therapy achieves a complete response (CR) rate of 22.2%, with a cumulative flare-free duration of 3.4 months.^[186]^ A prospective multicenter randomized trial conducted in China enrolled 195 adult ITP patients, comparing high-dose dexamethasone (HD-DXM group: 40 mg/d for 4 consecutive days) *versus* prednisone (PDN group: 1 mg/kg for 4 weeks with tapering). The HD-DXM group demonstrated superior overall initial response rate (82.1% *vs*. 67.4%, *P* = 0.044) and complete response rate (50.5% *vs*. 26.8%, *P* = 0.001).^[187]^

A systematic review incorporating 22 RCTs and 3 cohort studies (*n* = 1, 989) compared low-dose (LD) *versus* high-dose (HD) IVIG for pediatric ITP. Results showed no significant differences in the response rate (RR = 0.99, 95% CI: 0.96–1.02) or durable remission rate (defined as platelet count maintained within the normal range for at least 3 months without flares or additional drug treatment; RR= 0.97, 95% CI: 0.89–1.07). The two groups also exhibited similar results of time to platelet count elevation and time to hemostasis. Moreover, combined use with glucocorticoid (dexamethasone or methylprednisolone) could improve the therapeutic effect.^[188]^

For patients with inadequate response to high-dose glucocorticoid and/or IVIG, RTX may be considered. A multi-center retrospective study included 71 SLE patients with immune cytopenia. Before receiving RTX, these patients had received 3.1 ± 1.3 types of treatments, including glucocorticoids (100%) and hydroxychloroquine (88.5%). Among them, 44 patients with concurrent ITP received rituximab in addition to continued glucocorticoid combined with traditional immunosuppressant therapy, and the overall response rate (complete remission rate + partial remission rate) of these patients is 91%.^[156]^

Glucocorticoids combined with conventional immunosuppressants may prolong flare-free survival in patients. In a retrospective cohort study of 47 SLE patients with immune thrombocytopenia, after acute phase treatment with prednisone at 1 mg/kg/d, the patients received combination therapy with CTX, AZA, or RTX as induction therapy, respectively. CTX induction therapy was associated with a lower flare-free survival compared to RTX (CTX 43.6 weeks *vs*. RTX 51.8 weeks, *P* = 0.040) or AZA (CTX 43.6 weeks *vs*. AZA 51.2 weeks, *P* = 0.024).^[189]^ A retrospective cohort study of antinuclear antibody (ANA)-positive ITP patients revealed that the combination therapy of glucocorticoids and AZA achieved a significantly higher complete response rate (60.0% *vs*. 22.2%, *P* = 0.038) and a trend toward improved overall response rate (86.7% *vs*. 55.6%, *P* = 0.070). The combination therapy group also had a significantly prolonged cumulative flare-free survival (median: 7.8 *vs*. 3.4 months, *P* = 0.038). Multivariate analysis confirmed that combination therapy was independently associated with longer cumulative flare-free survival.^[186]^

In a multicenter, open-label, RCT of 120 immune thrombocytopenia patients with a maximum follow-up duration of 2 years, glucocorticoids combined with MMF showed a lower failure rate (RR = 0.41, 95% CI: 0.21–0.80; *P* = 0.008) and a greater complete response rate (platelet count >100×10^9^/L: 91.5% *vs*. 63.9%, *P* < 0.001) compared to glucocorticoid monotherapy. No significant differences were observed in bleeding events or treatment-related adverse effects (including infections) between the two groups.^[190]^

Combined therapy of glucocorticoids and tacrolimus (1 mg twice daily) was evaluated in a retrospective study of 20 SLE patients with immune thrombocytopenia. At 1 month, 3 patients (15%) achieved complete response, 14 (75%) achieved partial response (PR), and 3 (15%) were non-responders. At 3 months, all patients showed significant platelet improvements, with CR rate increasing to 25% (5 patients) and PR rate to 75%. At 6 months, all patients achieved sustained PR or CR without flare (CR: 75%).^[126]^

A retrospective study of 43 SLE-ITP patients (refractory to initial glucocorticoid/ immunosuppressants) compared between RTX and CsA with a follow-up period of at least 6 months. CsA could still induce remission in patients with prior treatment failure, but the remission rate was lower than that of patients treated with RTX.^[191]^

A Chinese single-arm, open-label trial of 14 SLE patients with refractory thrombocytopenia treated with sirolimus for 6 months reported an overall response rate of 71.4% and CR rate of 64.3%.^[106]^

For patients with poor response to the above-mentioned medications, or in emergency situations requiring rapid platelet elevation (*e.g*., life-threatening bleeding events or emergency surgery), thrombopoietin agents (*e.g*., recombinant human TPO [rhTPO], TPO receptor agonists [TPO-RA]) may be used. However, high-quality evidence for SLE-associated thrombocytopenia remains limited, and clinical practice mainly refers to ITP protocols. A multicenter open-label RCT in China comparing RTX+rhTPO *versus* RTX monotherapy in glucocorticoid-refractory/relapsed ITP demonstrateed that the combined therapy had a superior overall response rate (79.2% *vs*. 71.1%, *P* = 0.36) and complete response rate (45.4% *vs*. 23.7%, *P* = 0.026) compared with monotherapy.^[192]^ TPO-RAs including eltrombopag olamine and romiplostim have been approved for refractory ITP with favorable safety and efficacy profiles, and are recommended as second-line options in guidelines.

Splenectomy may be considered for SLE-ITP patients who cannot be effectively controlled by glucocorticoids and the aforementioned treatments or have persistent flares, though thrombocytopenia recurrence can still occur after splenectomy.^[193]^

**Question 7.3: How to diagnose and treat antiphospholipid syndrome (APS) in SLE?**

**Recommendation 7.3: It is recommended to refer to the 2023 ACR/EULAR APS classification criteria for diagnosis (1A). For SLE patients with thrombotic APS, long-term oral vitamin K antagonist anticoagulation therapy is recommended after the first arterial or unprovoked venous thrombotic event (1A). For patients with persistent positivity of antiphospholipid antibodies (APLs) but no thrombotic events, low-dose aspirin (75–100 mg/d) may be considered for primary prophylaxis (2B). For APLs-positive pregnant patients, low-dose aspirin and/or low molecular weight heparin (LMWH) are recommended, with stratified treatment based on prior pregnancy morbidity history, thrombotic history, and types of APLs (2B).**

The APLs included in the APS classification criteria mainly consist of anticardiolipin antibody (ACL), anti-β2 glycoprotein I antibody, and lupus anticoagulant (LA). Multiple large-scale prospective cohort studies, including CSTAR, have shown that approximately 30%–45% of SLE patients are APLs positive. APLs can affect vascular endothelial cell function, activate platelets, activate the complement system, promote inflammation and oxidative stress, and interfere with the coagulation and fibrinolytic systems, ultimately leading to clinical manifestations in SLE patients such as arterial/venous thrombotic events, microangiopathy, pregnancy morbidity, and cardiac valve damage. The 2023 ACR/EULAR APS classification criteria^[194]^ have been validated in multiple cohort studies to have very high specificity (95%–99%), and it is recommended to use these criteria in combination with clinical manifestations for the diagnosis of SLE with APS. However, the sensitivity of these criteria is only 81%–84%.^[195,196]^ Therefore, clinical diagnosis of SLE with APS requires comprehensive consideration of the patient’s overall clinical presentation and laboratory findings.

Long-term adequate anticoagulation remains the cornerstone of thrombotic APS treatment. A randomized and double-blind controlled trial published in 2003 involving 114 thrombotic APS patients demonstrated that long-term oral warfarin anticoagulation with a target international normalized ratio (INR) of 2.0–3.0 had comparable efficacy in thrombotic recurrence rates and similar bleeding risks to high-intensity warfarin anticoagulation (INR 3.1–4.0).^[197]^ For high-risk thrombotic APS patients, particularly those with arterial thrombosis, warfarin is recommended over direct oral anticoagulants (DOACs). An open-label randomized controlled trial published in 2018 compared rivaroxaban (20 mg/d) with warfarin (INR 2.0–3.0) in high-risk thrombotic APS patients. This study is the first to demonstrate that the rivaroxaban group has a significantly higher thrombotic recurrence rate (12%, including 4 ischemic strokes and 3 myocardial infarctions) compared to the warfarin group.^[198]^ A meta-analysis of DOACs in thrombotic APS published in 2023 showed that the use of DOAC was associated with an increased risk of arterial thrombotic events (OR = 5.43), particularly stroke (OR = 10.74), and an overall increase in arterial/venous thrombotic events (OR = 4.46).^[199]^ For APLspositive SLE patients without prior thrombotic events but with high-risk APL profiles (LA positivity, two APLs [any combination of LA, ACL, or anti-β2 glycoprotein I antibodies], or triple APLs positivity), low-dose aspirin (75–100 mg/d) should be considered for primary prophylaxis (Table 5).

**Table 5:** Treatment Recommendations for SLE with APL Positivity

Patient Population	Recommendation	Evidence Level	Recommendation Grade
Thrombotic Event Management			
APLs-positive SLE patients without symptoms	Aspirin (75–100 mg/d) for primary prophylaxis	2a	B
First unprovoked venous thrombosis	Long-term anticoagulation with vitamin K antagonist (INR 2.0–3.0)	2b	B
First arterial thrombosis in confirmed APS	Vitamin K antagonist preferred over aspirin alone; individualized INR targets (2.0–3.0 or 3.0–4.0) based on bleeding/thrombosis risk; considering warfarin plus aspirin	2b	C
Triple-APLs-positive patients with thrombosis	Rivaroxaban not recommended	1b	B
Pregnancy Morbidity Management			
APL-positive SLE patients without prior thrombosis/pregnancy morbidity	Aspirin (75–100 mg/d)	1b	B
APL-positive SLE patients with prior thrombosis	Aspirin combine with therapeutic-dose LMWH	1b	B
APL-positive SLE patients with recurrent early miscarriage meeting the APS classification criteria	Aspirin combine with prophylactic-dose LMWH	2b	B
APL-positive SLE patients with late miscarriage or pathological pregnancy related to placental insufficiency meeting the APS classification criteria	Aspirin combine with therapeutic-dose LMWH	2b	B

A small subset of APS patients may develop catastrophic APS (CAPS), characterized by multiple (≥3) organ thromboses within one week, involving critical organs such as the brain, kidneys, liver, or heart, leading to organ failure and death. The incidence of CAPS is approximately 1.0%, but the mortality rate is as high as 50%–70%. The pathogenesis may involve acute thrombotic storms and inflammatory cascades. High-quality evidence for the treatment of SLE with CAPS is lacking, but early diagnosis and aggressive treatment are critical.^[200,201]^ Early intervention for infections and avoidance of anticoagulation interruption or dose reduction may help prevent CAPS. First-line treatment for CAPS includes heparin anticoagulation combined with glucocorticoids, plasma exchange, and/or intravenous immunoglobulin,^[202]^ along with active identification and management of triggers such as infections or malignancies. Case reports and small-scale studies suggested that refractory CAPS may be treated with rituximab (B-cell depletion) or eculizumab (complement pathway blockade), but large-scale controlled studies are still lacking.^[203]^

APLs positivity is an independent risk factor for pregnancy morbidity in SLE patients.^[204]^ Multiple cohort studies on APS-related pregnancy outcomes have demonstrated that APLspositive patients have significantly higher rates of pregnancy complications, including recurrent early miscarriage, intrauterine fetal death, preeclampsia/eclampsia, gestational hypertension, fetal growth restriction, and preterm birth. During the perinatal period, SLE patients with persistent APLs positivity should be stratified based on prior thrombotic history, pregnancy morbidity history, and types of APLs to develop individualized treatment plans, with low-dose aspirin and/or LMWH recommended. A systematic review of 11 studies (including 9 RCTs and 2 quasi-RCTs) involving 1672 APLs-positive women (including SLE patients) showed that treatment with aspirin and heparin (unfractionated heparin or LMWH) during pregnancy significantly increased live birth rates compared to placebo or aspirin alone (RR = 1.27, 95% CI: 1.09–1.49), with no serious adverse effects or teratogenicity.^[205]^

**Question 7.4: How to treat neuropsychiatric SLE (NPSLE)?**

**Recommendation 7.4: For confirmed active NPSLE, glucocorticoids combined with immunosuppressants are recommended (1B). For patients with persistent APLs or cerebrovascular disease, combined antiplatelet and/or anticoagulant therapy should be considered (2B).**

The treatment principles and goals of NPSLE should follow the general principles and goals of SLE treatment. The treatment regimen includes glucocorticoids combined with immunosuppressants, antiplatelet/anticoagulant therapy, and symptomatic management.

For induction therapy of active NPSLE, glucocorticoids combined with immunosuppressants are currently recommended.^[12]^ For patients with mild to moderate NPSLE (*e.g*., mild cognitive dysfunction and headache without definitive organic neurological damage), the recommended glucocorticoid dose is prednisone 0.5–1 mg/kg/d or equivalent. For severe NPSLE with inflammatory manifestations (*e.g*., myelopathy, acute confusional state, and status epilepticus), early high-dose methylprednisolone pulse therapy (250–1000 mg/d intravenously for 3–5 days) is recommended.^[206]^

A systematic review reported that immunosuppressants used for NPSLE treatment include CTX, MMF, and AZA.^[110]^ In severe NPSLE patients, high-dose methylprednisolone pulse therapy combined with intravenous CTX improved psychiatric symptoms, with a total improvement rate of 94.7%, significantly superior to methylprednisolone pulse therapy alone (46.2%).^[207]^ A post-hoc subgroup analysis of an RCT comparing MMF and IV-CTX in non-renal SLE patients showed no statistical difference in achieving NPSLE remission between the two treatments. ^[208]^ After NPSLE reaches remission, maintenance therapy with low-dose glucocorticoids (≤5 mg/d prednisone or equivalent) combined with MMF or AZA is recommended.^[209]^

Multiple clinical studies and systematic reviews have reported that rituximab (monotherapy or combined with CTX) is effective for refractory NPSLE.^[155,210,211]^ A post-hoc analysis of two phase 3 studies on belimumab involving 45 mild NPSLE patients demonstrated that belimumab improved headache symptoms. ^[212]^ However, a pooled analysis of five clinical studies on belimumab showed no clear protective effect against NPSLE flares. ^[213]^ The therapeutic effects of telitacicept and anifrolumab in NPSLE remain unproven.

Observational studies have identified antimalarials as protective factors against NPSLE, particularly against epileptic seizures.^[214]^ Intrathecal injection of dexamethasone and MTX for NPSLE treatment is associated with higher survival rates and flare-free survival rates. In the subgroup of NPSLE patients with elevated cerebrospinal fluid protein levels, intrathecal injection of dexamethasone and MTX significantly improved prognosis (*P* < 0.001) ,^[215]^ but attention should still be paid to the neurotoxicity of MTX in sensitive individuals. A systematic review indicateed that IVIG and plasma exchange may be effective for inducing clinical remission of NPSLE.^[110]^

For NPSLE patients with persistent APLs positivity, both Chinese and international guidelines recommend antiplatelet therapy as primary prophylaxis to reduce thrombotic events.^[216]^ For thrombotic cerebrovascular disease, standard-intensity vitamin K antagonist therapy (target INR 2.0–3.0) should be initiated as secondary prevention.

For NPSLE patients with epilepsy, treatment with valproate, lamotrigine, or levetiracetam is recommended. Diazepam is the first-line treatment for terminating status epilepticus.^[217]^ Antianxiety/antidepressant medications and psychological interventions can effectively improve psychiatric symptoms in NPSLE patients.^[218]^ Meanwhile, prevention and identification of nervous system infections, blood pressure management, and metabolic factor monitoring are also essential.

**Question 8: What other therapeutic measures are available for SLE?**

**Recommendation 8: Plasma exchange or immunoadsorption may be considered as adjunctive therapy for severe or refractory SLE (2C). Intravenous immunoglobulin (IVIG) may be added for refractory SLE or SLE complicated with infections (2D).**

Plasma exchange and immunoadsorption (clearance of circulating immunoglobulin and immunocomplexes) may provide short-term clinical improvements in severe or refractory SLE and serve as an adjuvant to first-line therapies.^[110,219]^ A real-world retrospective study suggested that whole blood exchange therapy helped increase hemoglobin levels more rapidly in refractory autoimmune hemolytic anemia.^[220]^ A meta-analysis of 18 RCTs (1990–2020, *n*= 457, Chinese SLE patients) comparing immunoadsorption (IAS) *versus* non-IAS showed that IAS combined with medications reduced serum IgG, creatinine, anti-nuclear antibodies, urinary protein, and tumor necrosis factor-alpha (TNF-α) levels, while increased levels of complement C3 and C4. However, IAS groups had higher risks of fever or chills, hypotension, and bleeding.^[221]^

IVIG may benefit patients with refractory SLE or SLE complicated with infections.^[222]^ For LN unresponsive to induction therapy, IVIG demonstrated response rates of 60%–70% (excluding class V LN).^[223]^

Janus kinase inhibitors (JAKi), by blocking the JAK-STAT signaling pathway, disrupt cytokine-mediated signaling, thereby mitigating inflammation and immune dysregulation. JAKi hold significant potential for SLE treatment, particularly in patients refractory to conventional therapies. A meta-analysis of JAKi for SLE demonstrated superior efficacy over placebo, especially in improving musculoskeletal and mucocutaneous manifestations. JAKi-treated patients showed higher rates of achieving SRI-4 (risk ratio [RR] = 1.18, 95% CI: 1.07–1.31; *P =* 0.001), BICLA response (RR= 1.16, 95% CI: 1.02–1.31; *P =* 0.02), LLDAS attainment (RR = 1.28, 95% CI: 1.07–1.54; *P =* 0.008), and remission of arthritis and rash in SLEDAI-2000 (RR= 1.09, 95% CI: 1.00–1.18; *P =* 0.04), with similar adverse event rates to placebo (RR= 1.01, 95% CI: 0.97–1.04; *P =* 0.65).^[224]^ A phase II RCT (BRAVE I) of baricitinib (4 mg) in SLE revealed that 70 out of 104 patients (67%) achieved remission of arthritis and rash at week 24 (OR = 1.8 *vs*. placebo, 95% CI: 1.0–3.3; *P =* 0.0414), which significantly improved symptoms in patients with active SLE inadequately controlled by standard therapy, whereas baricitinib at a dose of 2 mg showed no benefits. The phase III SLE-BRAVE-I trial published in *The Lancet* in 2023 confirmed a superior SRI-4 response rate with baricitinib at a dose of 4 mg (*P =* 0.016).^[225]^ However, another phase III RCT published in *The Lancet* in 2023 failed to replicate these findings.^[226]^ Case reports and case series highlighted the efficacy of tofacitinib in SLE-associated cutaneous lesions, alopecia, and arthritis,^[227, 228, 229]^ and the efficacy of baricitinib in cutaneous manifestations and alopecia.^[230, 231, 232]^ Emerging data suggest that novel JAKi (*e.g*., deucravacitinib^[233]^ and filgotinib^[234])^ may also have efficacy for SLE, warranting further clinical exploration. Collectively, JAKi represent a potential therapeutic option, particularly for SLE with prominent cutaneous or musculoskeletal involvement.

Additional study suggests that tripterygium glycosides combined with conventional therapy may reduce lupus activity and improve overall response rate.^[235]^ However, the overall incidence of reproductive toxicity in patients using tripterygium glycosides is 17.9% (95% CI: 14.1%–22.5%),^[236]^ necessitating caution in clinical practice.

In recent years, genetically engineered precision cell therapies, particularly chimeric antigen receptor T cells (CAR-T), have shown preliminary success in treating refractory SLE. Case reports and case series indicated that CD19-targeted autologous CAR-T cells can effectively eliminate pathogenic autoantibodies, markedly reduce proteinuria levels, and improve renal function in refractory LN, thereby achieving low disease activity or clinical remission.^[237, 238, 239]^ In refractory SLE-associated thrombocytopenia, CAR-T therapy has demonstrated significant platelet count increase, enabling glucocorticoids and immunosuppressants tapering or discontinuation.^[240]^ A phase I trial of dual-targeting CD19 and B-cell maturation antigen (BCMA) autologous CAR-T cells in refractory SLE patients also reported favorable efficacy and safety profiles.^[241]^ However, challenges such as complex manufacturing processes, high treatment costs, uncertainties regarding long-term efficacy, safety, and optimal therapeutic protocols necessitate further high-quality clinical investigations.

**Question 9: How to select and use non-pharmacological therapies for SLE?**

**Recommendation 9: Non-pharmacological therapies for SLE patients aim to improve health-related quality of life and should follow these principles: (1) moderate exercise; (2) social and psychological interventions; (3) avoidance of common hazardous substances; (4) sun protection; (5) smoking cessation; and (6) vitamin D supplementation (1C).**

Non-pharmacological therapies for SLE patients aim to improve health-related quality of life. Exercise interventions were shown to reduce depression (standardized mean difference [SMD] = -0.40, 95% CI: -0.71 to-0.09) and fatigue (mean difference [MD] = -0.52, 95% CI: -0.91 to-0.13).^[242,243]^ Psychological interventions have been found to decrease anxiety (SMD = -0.95, 95% CI: -1.57 to-0.34), mental stress (SMD = -0.63, 95% CI: -1.02 to-0.23), and depression (SMD = -1.14, 95% CI: -1.84 to-0.44), and also aid in the control of disease activity (SMD = -0.34, 95% CI: -0.57 to-0.11).^[244]^ The skin is the most commonly affected organ in SLE, and certain cosmetics may contain substances that exacerbate lupus activity.^[245]^ Additionally, SLE patients should avoid exposure to hair dyes and eyebrow dyes.^[246,247]^ Ultraviolet (UV) light exposure can trigger SLE flares, and sun protection measures (*e.g*., sunscreen) can prevent UV-induced skin irritation and reduce skin inflammation,^[248, 249, 250]^ thereby decreasing the risk of disease flares. Compared to non-smokers, smokers have a higher risk of SLE (OR = 1.49, 95% CI: 1.06 to2.08), a higher score of SLEDAI (15.6 ±7.8 *vs*. 9.0 ±5.8), and a poorer response to pharmacological treatments (OR = 0.53, 95% CI: 0.31 to0.93).^[251,252]^ Osteoporosis is a major comorbidity in SLE patients, and their serum vitamin D levels are significantly lower than those in healthy individuals. Therefore, vitamin D supplementation may alleviate inflammation and reduce disease activity in SLE patients.^[253,254]^

**Question 10: How to prevent and control infections in SLE?**

**Recommendation 10: Timely assessment of infection risk in SLE patients is essential, and infections should be promptly prevented, identified, and managed (1B).**

In China, the proportion of SLE patients dying from infections has been increasing annually, and infections have become the leading cause of death in SLE patients, with over 50% of patients dying due to infections.^[255]^ Key risk factors for infections in SLE patients include methylprednisolone pulse therapy (HR = 1.5, 95% CI: 1.1 to 2.1), the doses of glucocorticoid exceeding prednisone 7.5 mg/d (OR = 2.7, 95% CI: 1.3 to 5.3), disease activity (HR = 1.0, 95% CI: 1.0 to 1.0), long disease duration (HR = 1.2, 95% CI: 1.1 to 1.3), involvement of the hematological system (OR = 2.5, 95% CI: 1.3 to 4.9), and serositis (OR= 2.8, 95% CI: 1.3 to 5.7). Hydroxychloroquine has been shown to reduce the risk of severe infections (HR = 0.7, 95% CI: 0.5 to 1.0).^[128,256]^ The serum C-reactive protein ≥46.8 mg/L, procalcitonin ≥0.53 μg/L, and lymphocyte count ≤1.0×10^9^/L suggest a high likelihood of infection,^[257,258]^ which warrants timely assessment, prompt identification, and active treatment, along with measures to prevent infection.

**Question 11: How to manage SLE patients during the peri-gestational period?**

**Recommendation 11: SLE patients should prepare for pregnancy after meeting the necessary conditions and completing pre-pregnancy counseling and risk assessment. Pregnancy can be considered in SLE patients when meeting the following criteria: (1) The disease has been stable for at least 6 months; (2) oral prednisone ≤15 mg/d (or equivalent doses of non-fluorinated glucocorticoids); (3) discontinuation of teratogenic drugs for the required duration; (4) 24-hour urine protein <0.5 g/d; and (5) no signs of vital organ damage (2B). For pregnant SLE patients, an individualized follow-up plan should be developed by a multidisciplinary team, including rheumatologists and obstetricians, to closely monitor the disease activity, and fetal growth and development (1C). If there is no contraindication, hydroxychloroquine (HCQ) should be used throughout pregnancy (1B).**

To reduce pregnancy complications in women with SLE and achieve favorable pregnancy outcomes, adequate preparation before conception and strict monitoring of the disease during pregnancy are necessary. Women with SLE who have severe organ dysfunction and/or damage should be informed about the risks associated with pregnancy.^[259,260]^ Compared to patients with non-active LN, patients with active LN have significantly higher risks of disease flares during pregnancy (OR = 2.04, 95% CI: 1.21 to 3.45), preeclampsia (OR = 2.62, 95% CI: 1.36 to 5.05), fetal loss (OR = 4.90, 95% CI: 1.54 to 15.59), and preterm birth (OR = 4.26, 95% CI: 2.19 to 8.31).^[261]^ Patients who have been in remission for more than 6 months before pregnancy, with urine protein <0.5 g/d, no renal failure, and discontinuation of teratogenic immunosuppressants for more than 1 year, have markedly higher rates of full-term delivery (76.47% *vs*. 23.08%) and live births (80.39% *vs*. 30.77%) compared to patients with active disease during the 6 months before pregnancy. They also have significantly lower risks of gestational hypertension (17.65% *vs*. 23.08%) and preeclampsia or eclampsia (9.80% *vs*. 15.38%).^[262]^

Pre-pregnancy counseling is crucial for successful pregnancies in SLE patients, as planned pregnancies reduce the risks of disease flares and adverse pregnancy outcomes compared to unintended pregnancies.^[263,264]^ SLE can increase the risk of adverse pregnancy outcomes,^[265]^ so strict disease control is essential before pregnancy. A multidisciplinary team (including at least rheumatologists and obstetricians) should perform pre-pregnancy assessment, strictly evaluate indications and contraindications for pregnancy, and closely monitor disease activity throughout pregnancy to achieve favorable maternal and fetal outcomes.^[266,267]^ Specific measures for multidisciplinary management of SLE pregnancies by rheumatologists and obstetricians are detailed in the “2022 Chinese guideline for the management of pregnancy and reproduction in systemic lupus erythematosus”.^[268]^

Close monitoring of disease activity and fetal growth and development in SLE patients during pregnancy is critical for favorable maternal and fetal outcomes. Screening risk factors associated with pregnancy-related complications, such as anti-phospholipid antibodies, should be conducted before pregnancy. During pregnancy, strict monitoring of disease activity, placental function, and fetal growth and development is essential.^[267,269, 270, 271, 272, 273]^

HCQ can reduce the risks of disease activity, disease flares, and adverse fetal outcomes such as preterm birth in SLE patients. Continuous HCQ therapy is associated with a lower incidence of SLE flares during and after pregnancy. Therefore, HCQ should be used continuously throughout pregnancy if no contraindications exist.

**Question 12: How to manage vaccination in SLE patients?**

**Recommendation 12: Vaccination is an effective measure to prevent infections in SLE patients. The feasibility and timing of vaccination for adult SLE patients should be determined based on disease activity, current treatment regimen, type of vaccines, and patient preferences (2D).**

Infections remain one of the leading causes of death in SLE patients. Glucocorticoids and cytotoxic immunosuppressive agents can interfere patients’ immune responses to viral and bacterial pathogens, increasing the risk of infections. Current evidence indicates that SLE patients generally show good immunogenicity and tolerance to vaccines, which can decrease the rates of emergency visits and hospitalization due to infections, as well as the occurrence of invasive infections. No evidence up to now suggests that vaccination increases the risk of disease flares. Therefore, multiple international guidelines recommend that rheumatologists should conduct a comprehensive assessment of vaccination status for SLE patients annually.^[274,275]^ The vaccination should be scheduled based on current disease activity, current glucocorticoid dosage, use of immunosuppressive agents or biologics (especially B-cell-depleting therapies), and patient preferences. In general, SLE patients receiving glucocorticoids and immunosuppressive agents can receive inactivated vaccines, but live-attenuated vaccines are not recommended.

Currently, vaccines recommended for SLE patients include influenza vaccines, pneumococcal vaccines, hepatitis B vaccines, human papillomavirus vaccines, and herpes zoster vaccines. All of these vaccines available in China are inactivated or polysaccharide/recombinant protein formulations. Detailed vaccination indications and immunosuppressive drug adjustments are detailed in Table 6.

**Table 6:** Vaccination for SLE Patients

Vaccination	Eligible Population	Adjustment of Immunosuppressive Therapy
Influenza Vaccine	Patients aged ≥65 years, and patients aged 18–65 years who are receiving immunosuppressive therapy.	Discontinue MTX for 2 weeks after vaccination.
Pneumococcal Vaccine	Patients aged <65 years who are receiving immunosuppressive therapy.	If receiving glucocorticoids equivalent to prednisone ≥20 mg/d, delay vaccination until the dose is reduced to < 20 mg/d.
Hepatitis B Vaccine	Patients susceptible to hepatitis B virus.	Delay rituximab administration for 2 weeks after vaccination.
HPV Vaccine	Patients aged 9–45 years who are receiving immunosuppressive therapy and have not been previously vaccinated for HPV.	
Recombinant Herpes Zoster Vaccine	Patients aged >18 years who are receiving immunosuppressive therapy.	

a For patients receiving glucocorticoids equivalent to prednisone <20 mg/d, if vaccination is critical and discontinuation of glucocorticoids pose high risks of disease flares or adrenal insufficiency, continuation of low-dose glucocorticoids may be considered. ^b^For patients receiving methotrexate ≤0.4 mg/kg/week or azathioprine ≤3 mg/kg/d (low-level immunosuppressive therapy), if vaccination is critical and discontinuation of immunosuppressants pose high risks of disease flares, the duration of drug discontinuation may be shortened. HPV, human papillomavirus.

## Lead Experts

Xiaofeng Zeng (Department of Rheumatology and Immunology, Peking Union Medical College Hospital, Chinese Academy of Medical Sciences & Peking Union Medical College); Mengtao Li (Department of Rheumatology and Immunology, Peking Union Medical College Hospital, Chinese Academy of Medical Sciences & Peking Union Medical College).

## Lead Methodology Expert

Yaolong Chen (Innovation Unit of Evidence-Based Evaluation and Guidelines, Chinese Academy of Medical Sciences; Evidence-Based Medicine Center, School of Basic Medical Sciences, Lanzhou University).

## Steering Committee

Xiaofeng Zeng (Department of Rheumatology and Immunology, Peking Union Medical College Hospital, Chinese Academy of Medical Sciences & Peking Union Medical College); Mengtao Li (Department of Rheumatology and Immunology, Peking Union Medical College Hospital, Chinese Academy of Medical Sciences & Peking Union Medical College); Yaolong Chen (Innovation Unit of Evidence-Based Evaluation and Guidelines, Chinese Academy of Medical Sciences; Evidence-Based Medicine Center, School of Basic Medical Sciences, Lanzhou University); Xinping Tian (Department of Rheumatology and Immunology, Peking Union Medical College Hospital, Chinese Academy of Medical Sciences & Peking Union Medical College); Jiuliang Zhao (Department of Rheumatology and Immunology, Peking Union Medical College Hospital, Chinese Academy of Medical Sciences & Peking Union Medical College).

## Evidence Evaluation Group and Secretariat

Yinghua Chen (Department of Nephrology, Eastern Theater General Hospital); Yisha Li (Department of Rheumatology and Immunology, Xiangya Hospital, Central South University); Tingting Liu (Department of Rheumatology and Immunology, Ruijin Hospital, Shanghai Jiao Tong University School of Medicine); Shangzhu Zhang (Department of Rheumatology and Immunology, Peking Union Medical College Hospital, Chinese Academy of Medical Sciences & Peking Union Medical College); Ting Zhang (Department of Rheumatology and Immunology, The Second Affiliated Hospital, Zhejiang University School of Medicine); Mengmeng Zhao (Department of Rheumatology and Immunology, The First Affiliated Hospital of China Medical University); Yangzhong Zhou (Department of Rheumatology and Immunology, Peking Union Medical College Hospital, Chinese Academy of Medical Sciences & Peking Union Medical College); Ling Wang (School of Population Medicine and Public Health, Peking Union Medical College); Yuanyuan Yao (School of Basic Medical Sciences, Lanzhou University); Ye Wang (School of Public Health, Lanzhou University); Zhewei Li (School of Public Health, Lanzhou University); Bingyi Wang (School of Basic Medical Sciences, Lanzhou University).

## Expert Panel (Listed in Alphabetical Order by Surname)

Shuhong Chi (Department of Rheumatology and Immunology, General Hospital of Ningxia Medical University); Zhanyun Da (Department of Rheumatology and Immunology, Affiliated Hospital of Nantong University); Shengming Dai (Department of Rheumatology and Immunology, Shanghai Sixth People’s Hospital); Xinwang Duan (Department of Rheumatology and Immunology, The Second Affiliated Hospital of Nanchang University); Lingli Dong (Department of Rheumatology and Immunology, Tongji Hospital, Tongji Medical College, Huazhong University of Science and Technology); Xuebing Feng (Department of Rheumatology and Immunology, Nanjing Drum Tower Hospital, Nanjing University Medical School); Jinsong Gao (Department of Obstetrics and Gynecology, Peking Union Medical College Hospital, Chinese Academy of Medical Sciences & Peking Union Medical College); Lan He (Department of Rheumatology and Immunology, The First Affiliated Hospital of Xi’an Jiaotong University); Wenhui Huang (Department of Rheumatology and Immunology, The Second Affiliated Hospital of Guangzhou Medical University); Lindi Jiang (Department of Rheumatology and Immunology, Zhongshan Hospital, Fudan University); Zhenyu Jiang (Department of Rheumatology and Immunology, The First Hospital of Jilin University); Hongzhong Jin (Department of Dermatology, Peking Union Medical College Hospital, Chinese Academy of Medical Sciences & Peking Union Medical College); Caifeng Li (Department of Rheumatology and Immunology, Beijing Children’s Hospital, Capital Medical University); Fen Li (Department of Rheumatology and Immunology, The Second Xiangya Hospital of Central South University); Hongbin Li (Department of Rheumatology and Immunology, Affiliated Hospital of Inner Mongolia Medical University); Mengtao Li (Department of Rheumatology and Immunology, Peking Union Medical College Hospital, Chinese Academy of Medical Sciences & Peking Union Medical College); Juan Li (Department of Rheumatology and Immunology, Nanfang Hospital, Southern Medical University); Xiaomei Li (Department of Rheumatology and Immunology, The First Affiliated Hospital of University of Science and Technology of China); Jin Lin (Department of Rheumatology and Immunology, The First Affiliated Hospital, Zhejiang University); Shudian Lin (Department of Rheumatology and Immunology, Hainan General Hospital); Shunping Lin (Department of Rheumatology and Immunology, Fujian Medical University Union Hospital); Zhiming Lin (Department of Rheumatology and Immunology, The Third Affiliated Hospital of Sun Yat-sen University); Dongzhou Liu (Department of Rheumatology and Immunology, Shenzhen People’s Hospital); Shengyun Liu (Department of Rheumatology and Immunology, The First Affiliated Hospital of Zhengzhou University); Yi Liu (Department of Rheumatology and Immunology, West China Hospital, Sichuan University); Hui Luo (Department of Rheumatology and Immunology, Xiangya Hospital, Central South University); Liangjing Lyu (Department of Rheumatology and Immunology, Renji Hospital, Shanghai Jiao Tong University School of Medicine); Hanyou Mo (Department of Rheumatology and Immunology, The First Affiliated Hospital of Guangxi Medical University); Haili Shen (Department of Rheumatology and Immunology, The Second Hospital of Lanzhou University); Xiaofei Shi (Department of Rheumatology and Immunology, The First Affiliated Hospital of Henan University of Science and Technology); Qiang Shu (Department of Rheumatology, Qilu Hospital of Shandong University); Yijun Song (Department of Obstetrics and Gynecology, Peking Union Medical College Hospital, Chinese Academy of Medical Sciences & Peking Union Medical College); Yin Su (Department of Rheumatology and Immunology, Peking University People’s Hospital); Lingyun Sun (Department of Rheumatology and Immunology, Nanjing Drum Tower Hospital, Nanjing University Medical School); Wenfeng Tan (Department of Rheumatology and Immunology, The First Affiliated Hospital of Nanjing Medical University); Xinping Tian (Department of Rheumatology and Immunology, Peking Union Medical College Hospital, Chinese Academy of Medical Sciences & Peking Union Medical College); Caihong Wang (Department of Rheumatology and Immunology, The Second Hospital of Shanxi Medical University); Jing Wang (Department of Rheumatology and Immunology, Yunnan Provincial First People’s Hospital); Qian Wang (Department of Rheumatology and Immunology, Peking Union Medical College Hospital, Chinese Academy of Medical Sciences & Peking Union Medical College); Yongfu Wang (Department of Rheumatology and Immunology, The First Affiliated Hospital of Baotou Medical College, Inner Mongolia University of Science and Technology); Wei Wei (Department of Rheumatology and Immunology, Tianjin Medical University General Hospital); Zhenbiao Wu (Department of Rheumatology and Immunology, The Second Affiliated Hospital of Air Force Medical University); Lijun Wu (Department of Rheumatology and Immunology, People’s Hospital of Xinjiang Uygur Autonomous Region; Xinjiang Clinical Research Center for Rheumatoid Arthritis); Jian Xu (Department of Rheumatology and Immunology, The First Affiliated Hospital of Kunming Medical University); Jing Xue (Department of Rheumatology and Immunology, The Second Affiliated Hospital, Zhejiang University School of Medicine); Chengde Yang (Department of Rheumatology and Immunology, Ruijin Hospital, Shanghai Jiao Tong University School of Medicine); Min Yang (Department of Rheumatology and Immunology, Nanfang Hospital, Southern Medical University); Niansheng Yang (Department of Rheumatology and Immunology, The First Affiliated Hospital of Sun Yat-sen University); Pingting Yang (Department of Rheumatology and Immunology, The First Affiliated Hospital of China Medical University); Qibing Xie (Department of Rheumatology and Immunology, West China Hospital, Sichuan University); Jiashun Zeng (Department of Rheumatology and Immunology, Affiliated Hospital of Guizhou Medical University); Xiaofeng Zeng (Department of Rheumatology and Immunology, Peking Union Medical College Hospital, Chinese Academy of Medical Sciences & Peking Union Medical College); Liyun Zhang (Shanxi Bethune Hospital, Shanxi Academy of Medical Sciences); Miaojia Zhang (Department of Rheumatology and Immunology, The First Affiliated Hospital of Nanjing Medical University); Xiao Zhang (Department of Rheumatology and Immunology, The Eighth Affiliated Hospital of Sun Yat-sen University); Wen Zhang (Department of Rheumatology and Immunology, Peking Union Medical College Hospital, Chinese Academy of Medical Sciences & Peking Union Medical College); Zhuoli Zhang (Department of Rheumatology and Immunology, Peking University First Hospital); Dongbao Zhao (Department of Rheumatology and Immunology, The First Affiliated Hospital of Naval Medical University); Jiuliang Zhao (Department of Rheumatology and Immunology, Peking Union Medical College Hospital, Chinese Academy of Medical Sciences & Peking Union Medical College); Yan Zhao (Department of Rheumatology and Immunology, Peking Union Medical College Hospital, Chinese Academy of Medical Sciences & Peking Union Medical College); Yi Zhao (Department of Rheumatology and Allergy, Xuanwu Hospital, Capital Medical University); Jing Zhu (Department of Rheumatology and Immunology, Sichuan Provincial People’s Hospital).
